# Fused Deposition Modeling of Poly (lactic acid)/Macadamia Composites—Thermal, Mechanical Properties and Scaffolds

**DOI:** 10.3390/ma13020258

**Published:** 2020-01-07

**Authors:** Xiaohui Song, Wei He, Huadong Qin, Shoufeng Yang, Shifeng Wen

**Affiliations:** 1 College of Chemistry & Chemical Engineering and Guangxi Key Laboratory of Processing for Non-ferrous Metallic and Featured Materials, Guangxi University, Nanning 530004, China; songxiaohui2010@163.com; 2 College of Mechanical Engineering, Guilin University of Aerospace Technology, Guilin 541004, China; tanhd13@lzu.edu.cn; 3 Department of Mechanical Engineering, Katholieke Universiteit Leuven, PO box 2420, Heverlee, 3001 Leuven, Belgium; s.yang@soton.ac.uk; 4 Faculty of Engineering and Environment, University of Southampton, Southampton SO17 1BJ, UK; 5 State Key Laboratory of Materials Processing and Die and Mould Technology, School of Materials Science and Engineering, Huazhong University of Science and Technology, Wuhan 430074, China; roya_wen@hust.edu.cn

**Keywords:** fused deposition modeling, PLA polymer-matrix, macadamia nutshell, mechanical properties, thermal properties

## Abstract

In this work Macadamia nutshell (MS) was used as filler in fused deposition modeling (FDM) of Poly (lactic acid) (PLA) composites filaments. Composites containing MS both treated and untreated with alkali and silane were investigated by means of Fourier transform infrared spectroscopy (FTIR), X-Ray diffraction (XRD), Thermogravimetry (TG), scanning electron microscopy (SEM). The results showed that the treated MS composites had better thermal stability. Furthermore, compression tests were carried out. The PLA with 10 wt% treated MS composite was found possessing the best mechanical properties which was almost equivalent to that of the pure PLA. Finally, porous scaffolds of PLA/10 wt% treated MS were fabricated. The scaffolds exhibited various porosities in range of 30–65%, interconnected holes in size of 0.3–0.5 mm, micro pores with dimension of 0.1–1 μm and 37.92–244.46 MPa of elastic modulus. Those values indicated that the FDM of PLA/MS composites have the potential to be used as weight lighter and structural parts.

## 1. Introduction

As a promising technology, the Additive Manufacturing (AM) technology offers a new strategy for producing customized parts with desired architecture, shape, pores and porosity [1,2]. Based on the AM, the Fused deposition modeling (FDM) melts a spool of thermoplastic filament and extrudes it onto a platform to create tangible 3 dimensional parts [3]. Poly (lactic acid) (PLA) is the most extensively used material in FDM, and has been regarded as a promising material for its eco-friendly, biocompatible and processability [4]. However, its relatively hydrophobic and low degradable properties have limited its use [5].

Natural biomass is known as being hydrophilic, biodegradable and recyclable [6]. Driven by the increasing environmental pollution and global energy crisis, natural biomass has been used as the reinforcements on the polymer-matrix composites [7]. Some biomass has been successfully used for PLA-matrix, including seeds (e.g., cotton and milkweed), fruit nutshells (e.g., coconut shell and peanut shell), basts (e.g., flax, lamp and jute), leaves (e.g., sisal and banana) and grass/cane/reed fibers (e.g., bamboo) [8,9].

Some researchers have combined the study of FDM and the PLA/natural biomass composites. Tao et al. [10] prepared the PLA/5 wt% wood flour composites using the FDM and enhanced the initial deformation resistance of the composite. Ayrilmis et al. [11] studied the effects of the layer thickness on the water absorption and mechanical properties of the FDM PLA/30 wt% wood composites. Gkartzou et al. [12] processed the PLA/kraft lignin with FDM and found that the highest content ratio of kraft in composite was 15 wt%. Yu et al. [13] studied the internal morphology of the FDM PLA/basalt composites. Moreover, continuous fibers, such as jute and flax, were used on the FDM setup with PLA [12,14,15]. These studies demonstrated that the FDM of PLA/natural biomass is feasible and meaningful.

Macadamia nuts are mainly produced in Australia and are also produced in China and Latin American countries. Its total production is about 100,000 ton per year. The shell takes almost 70% weight of a macadamia nut. The bulk density of the milled MS powders is 0.54 g/cm^3^ [16], which enables to reduce the weight of the polymer-matrix composites. Few of them are used as active carbon [17]. However, most of them were ground to compost or thrown as waste [18], which strongly reduced its value.

Recently, there has been a growing interest of using the macadamia nutshells in composite materials. Dong investigated the mechanical properties of the PLA/macadamia composites [19]. Kumar studied the thermo-mechanical characterization of the PLA/macadamia composites with triacetin as a plasticizer [16]. Dong reinforced polyester using macadamia nutshells and carried out the flexural tests of the composites [20]. Those composites were prepared using the conventional methods, including injection molding and compression molding, which were hardly able to satisfy the requirements of the structural parts with internal and external structure. However, literatures about the application of FDM in the process of polymer/MS composites were very few. Only in 2016, Jordan explored the FDM of ABS/macadamia nutshells composites [21].

The aim of this study was to develop the FDM of the relatively cheap and weight-lighter PLA/MS composite filaments with acceptable mechanical properties. The study focused on: Investigating the chemical groups and crystal degree of the untreated and treated MS; the effect of MS content (5–15 wt%) on the melt flow index, the thermal and mechanical properties of composites; characterizing the microstructure of the fractured surface and fabricating the porous scaffolds with interconnected pores and controllable porosity.

## 2. Experimental Details and Characterization Methods

### 2.1. Materials

The used Poly (lactic acid) (PLA) matrix, with trade name 2002D, was supplied by NatureWorks LLC (Minnetonka, MN, USA) in the form of powder; the main characteristics provided by the supplier are: Density of 1.24 g/cm^−3^, melting temperature of 151°, Mn of 113,300 Da and Mw of 181,600 Da. Macadamia nutshells (MS) were collected from the discards of macadamia nut food with a density of 0.69 g/cm^−3^ measured by the pycnometer method. Before being further used, the MS were cleaned with purified water, and dried and ground into powders. These powders were then sifted by using a 300-mesh screen (~50 µm).

### 2.2. Experimental Procedure

**Figure 1:**
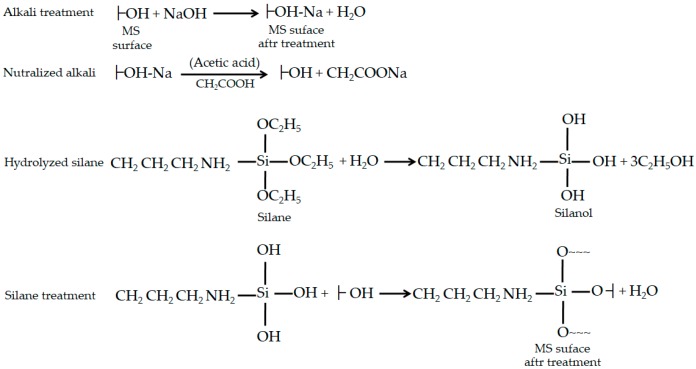
The reaction of alkali and silanol with MS.

The macadamia nutshells powder were modified: first, soaked in 5 wt% concentration of a aqueous sodium hydroxide at room temperature for 5 h and then washed until the pH level was 7; third, immersed in a 2–6 wt% concentration of a 3-Aminopropyltriethoxysilane (KH550) solution with a pH of 3.5–4, then continuously stirred with a magnetic stirring apparatus for 12 h. The reaction mechanism of the NaOH and KH550 with MS is shown in Figure 1 [22]. Finally, it was washed with deionized water and dried in a laboratory oven at 80 °C until the moisture content became 0–1% measured by using a scale with an error of 0.001.

The MS powder, including the untreated and treated ones, with as-received PLA powders were blended for 24 h by using a planetary ball mill (QM–3SP4, Nanjing Yifan Apparatus Co. Ltd., Nanjing, China). Zirconium balls of 5 mm and 10 mm diameter were added for improving the grinding homogeneity of mixture, the weight ratio between ball and material was 1:1 [23]. After being blended, the formulations of the PLA-MS composites are listed in Table 1.

Filaments with a diameter of 1.75 ± 0.3 mm were obtained from blended composite powders by using a customized desktop single screw extruder. The parameters were chosen as: 20 rpm for the screw speed, 165 °C for the barrel temperature, and 1.5 mm for the die diameter. Then the filaments were applied to a commercial 3D printer (Allct Yinke, Wuhan, China). The processing parameters were as: 50 mm/s for the printing speed, 210 °C for the nozzle heater temperature, 0.06 mm for the layer thickness, 100% for the fill ratio, 100% extrusion ratio, 0.4 mm for the shell thickness, and 45 °C for the platform temperature and a linear filling mode. Four cylindrically shaped compressive samples with a diameter of 10 mm and a height of 12 mm were printed based on the GBT 1041 standard [24].

### 2.3. Characterization Methods

The spectral changes of the surface functional groups of the treated MS were determined using a Fourier transform infrared spectrometer (FTIR) (Thermo Nicolet AVATAR FTIR 360, Thermo Nicolet Corporation, Madison, WI, USA) at room temperature. Infrared spectra of untreated and treated MS samples were analyzed in the 4000–400 cm^−1^ range.

The crystallinity and structural characterization of the MS powders were investigated by using a X-ray powder diffractometer (RigaKu D/MAX 2500V, RigaKu D/MAX 2500V, Rigaku Co., Tokyo, Japan) equipped with a Cu Kα radiation source (λ = 1.54060 Å), operating at a voltage of 40 kV and intensity of 40 mA, over the incidence angle (2θ) in the range of 5–50°, at the ambient temperature. The crystallinity index ($I_{c}$) was obtained with the empirical Segal equation [25]:

(1) $$I_{c}\left(\%\right)=\frac{I_{002}-I_{amp}}{I_{002}}\times 100\%$$

where $I_{002}$ is the intensity at 2θ = 22.1°, representing the crystalline material. $I_{amp}\;\mathrm{is}$ the intensity at 2θ = 16°, contributing to the amorphous material of MS.

A simultaneous thermal analyzer (Labsys Evolution1600, Setaram, France) was used to measure the thermal stability of the PLA/MS composites at a dry Nitrogen atmosphere. About 20 mg of composite was heated from room temperature to 900 °C at a rate of 10 °C/min and held for 5 min, and then cooled naturally.

According to the standard ASTM D1238-73 [26], the test of the Melt Flow Index (MFI) of the treated and untreated composites was carried out. The MFI was recorded in grams per 10 min (six times per sample) on a melt flow tester (Tiansu cablication, Shenzhen, China) with a capillary die with a standard diameter of 2.0955 ± 0.001 mm. The cylinder temperature was set as 165 °C and the load was 2.5 kg.

The compression properties of the PLA/MS samples were tested using a universal testing machine (Zwick Roell 2KN, Ulm, Germany,) at a rate of 1 mm/min and preload of 0.1 N. The brittle fracture surfaces of samples were characterized by using a scanning electron microscope (SEM, HITACHI SU8020 system, Tokyo, Japan). Prior to scanning, the fracture surface was gold coated.

The porosity of the scaffold manufactured by using FDM can be obtained by this equation [27]

(2) $$Porosity=1-\frac{\rho_{0}}{\lambda_{PLA}\times \rho_{PLA}+\lambda_{MS}\times \rho_{MS}}\;\;\;$$

where $\lambda_{PLA}$ and $\lambda_{MS}$ denote the weight ratio of PLA and MS in composites; $\rho_{PLA}$ and $\rho_{MS}$ are the theoretical densities (g/cm^3^) of PLA and MS, respectively; $\rho_{PLA}$ equals to 1.24 g/cm^3^ provided by the manufacturers; $\rho_{MS}$ is equal to 0.69 g/cm^3^ measured with the pycnometer method; $\rho_{0}$ denotes the apparent density computed through:

(3) $$\rho_{0}=m_{0}/V_{0}$$

where $m_{0}$ denotes the mass of the MS sample (g) gotten by using a scale with accuracy of 0.001 g, and $V_{0}$ is the sample volume (mm^3^) measured by using a Vernier caliper. The average density of four scaffolds was taken as the density of the PLA/MS composite.

## 3. Results and Discussion

### 3.1. Morphology of MS Particles

**Figure 2:**
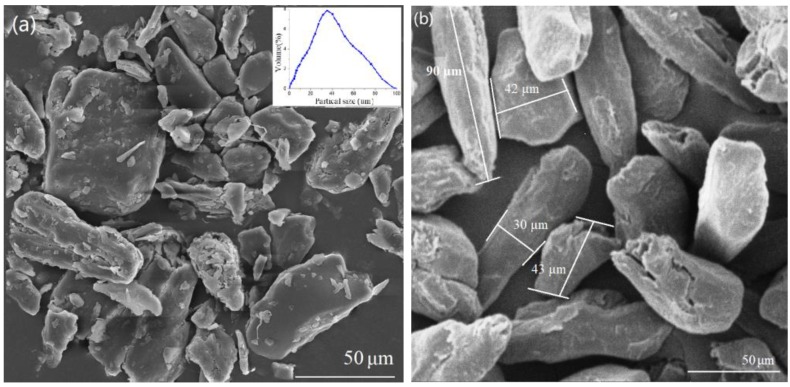
The morphology and distribution of MS particles: (**a**) before treated with NaOH/silane and (**b**) after treated with NaOH/silane.

Figure 2 shows the distribution size (tested with the wet dispersion method on a Mastersizer (MAZ 3000, Malvern Panalytical, Malvern, UK) and the morphology (examined with the scanning electron microscopy using the HITACHI SU8020 system) of the ground MS particles. The particles possess an irregular shape with an average size of about 40 µm. The smaller particles show the tendency to agglomerate together. The impurities adhere to the surface of the bigger MS particles. This might hamper the surface compatibility between PLA and MS. Figure 2b gives the morphology of the modified MS powders. It can be seen that the size of the MS particles is more uniform than that of the untreated ones. The dimension of the treated particles is in a range of 30–42 μm in width and 43–92 μm in length. The smaller impurities have been removed from the surface of the MS particles. There is no agglomeration between the treated MS particles, showing potential to be compatible with PLA polymer.

### 3.2. FTIR Analysis

**Figure 3:**
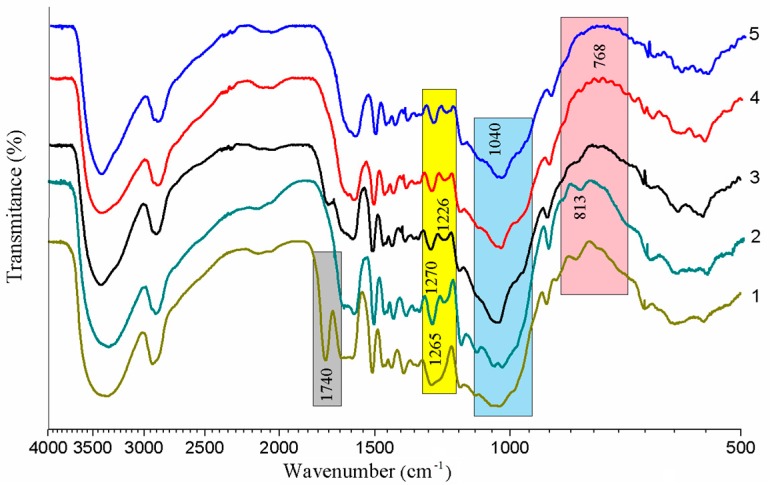
The FTIR spectra of: 1. MS; 2. NaOH treated MS; 3. NaOH + 2 wt% KH550 treated MS; 4. NaOH + 4 wt% KH550 treated MS; 5. NaOH + 6 wt% KH550 treated MS.

The FTIR spectra of the untreated and treated MS are presented in Figure 3. The spectra of the untreated MS are similar to that described in other literature [16]. The downward peak between 3500–3200 cm^−1^ representing the hydrogen bonded O-H groups [28] became narrower in the spectra of the treated MS, revealing that the ratio of the O-H in MS increased. The band at 1740 cm^−1^, due to the stretching vibration in carbonyl C = O groups [29], disappears after the alkali treatment. This showed that most of the hemicelluloses along with whole ash, oil, and other impurities have been removed from the MS. The band in sample 1 at 1265 cm^−1^, representing the C–O stretching vibrations in acetyl group of in lignin and hemicellulose, cleaved into two narrow peaks at 1270 cm^−1^ and 1226 cm^−1^ in the spectrum of the treated MS (samples 3–5). This result indicated that the rest of the hemicellulose had been cleared from the MS.

After treated with the KH550, the band at 1040 cm^−1^ was enhanced, especially in the sample 3, which can be attributed to the Si-O and Si-O-C stretching vibration [30]. Meanwhile, the peak at 813 cm^−^^1^, relevant to the C-H out-of-plane bending vibration, is fading away with the increasing of the KH550’s concentration. A small peak at the 768 cm^−1^ (sample 3 and sample 4) is probably due to a condensation reaction between the MS and the silanol.

### 3.3. XRD Analysis

**Figure 4:**
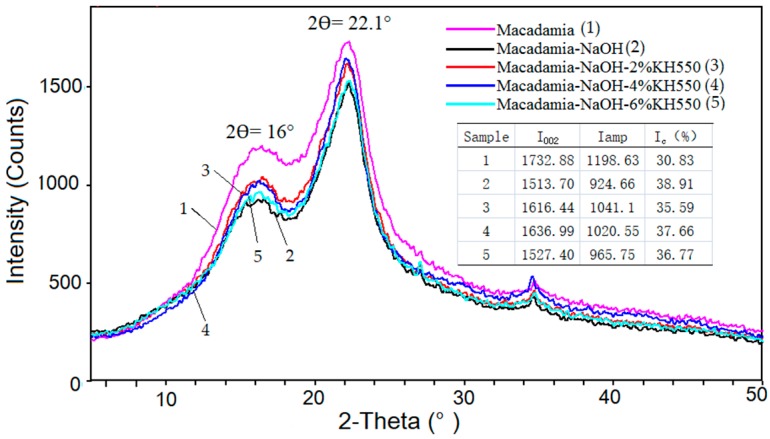
The X-Ray diffraction analysis of the untreated and treated MS ($I_{002}$ is the intensity at 2θ = 22.1°, $I_{amp}\mathrm{is}$ the intensity at 2θ = 16° and $I_{c}$ is the crystallinity index).

The X-ray powder diffraction (XRD) patterns of the untreated and treated MS are given in Figure 4. All the samples show a sharp peak at 2θ = 22.1°, representing the crystalline material, and a shoulder at 2θ = 16°, contributing to a typical form of cellulose I [31]. The crystallinity index ($I_{c}$) of the NaOH treated MS (38.91%) was enhanced due to the removing of the hemicelluloses, oil, pectin and other impurities from MS [26]. The cellulose chains would better repack after the alkali treatment. Similar results have been reported elsewhere [32,33]. The treatment with silane leads to a decline in $I_{c}$ of MS, when compared to that of the alkali treated MS. $I_{c}$ decreased to 35.59% at 2 wt% concentration of silane, and then goes up again with the content increasing of silane. The decreasing crystallinity index of biomass after silane treatment has been reported in other studies [25,32]. The introduced silane reacted with MS, resulting in the O-H groups’ cleavage of MS, which made the transformation of crystal region in MS to its amorphous region. When the KH550 reached a certain concentration level (4 wt%), the abundant silane stopped to react with the MS and was physically tangled with MS, resulting in the climbing of $I_{c}$ of MS.

### 3.4. The Thermal Stability of the Pure PLA and Its Composites

**Figure 5:**
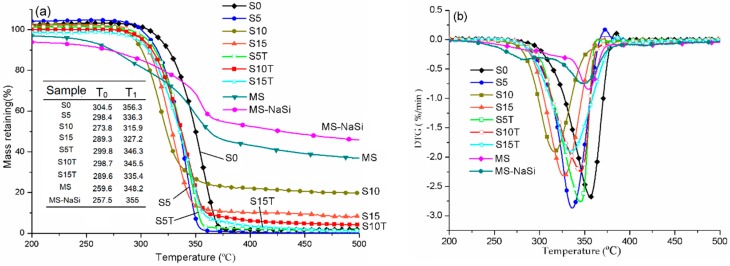
The thermal stability of PLA, MS and their composites: (**a**) TG curves and (**b**) DTG curves. (T_0_ is the onset temperature of degradation, T_1_ is the temperature at maximum degradation rate).

According to the previous analysis, 2 wt% of silane performed the best during the treatment of the MS. Therefore, this was chosen to develop some further experiments. The thermal stability of the pure PLA, MS and the PLA/MS composites are depicted and summarized in Figure 5. The temperature window between 200 and 500 °C is shown since no significant changes in the TG and DTG curves below 200 °C and between 400 and 800 °C have been observed.

The TG curves of the pure PLA, MS and all their composites exhibit a single step thermal degradation. The degradation of MS and MS-NaSi started at 250 °C approximately, got to the maximum degradation rate at about 350 °C and basically completed by around 400 °C, fowling by a slow mass loss until to the final temperature. The DTG curve of MS produces a major peak at 348.2 °C and a shoulder peak at 281 °C, which can contribute to the degradation of cellulose and hemicelluloses, respectively. It has been well known that the MS consists of 29.5% of cellulose, 30% of hemicellulose, 40.1% of lignin, along with some impurities [18]. Meanwhile, reference [34] showed that the degradation temperature of the hemicelluloses is in a range of 220–315 °C. After the NaSi treatment, the lower peak of hemicellulose disappeared from the DTG curve of MS-NaSi, indicating that the hemicellulose has been removed from the MS by chemical treatment. The TG and DTG curves of MS-NaSi shift to the higher temperature, showing that the MS-NaSi was more thermal stable than MS. However, both MS and MS-NaSi degraded at a level less than 1.0 min^−1^, indicating that they are less reactive.

PLA possessed the highest onset temperature of degradation (T_0_) and the temperature at maximum degradation rate (T_1_) with values of 304.5 °C and 356.3 °C at a level of 2.75 min^−^^1^. The incorporation of MS reduced the T0 and T1 of composites, when compared to that of PLA. Both T0 and T1 of sample S10 showed the biggest reduction with 273.8 °C and 315.9 °C, respectively. The hemicellulose in the MS reduced the degradation temperature of PLA/MS composites. Meanwhile, the impurities in the MS strongly affected the interfacial compatibility between the MS particles and the PLA matrix, resulting in a weaker thermal stability.

After incorporation of alkali/silane, the thermal stability of the treated samples was improved dramatically. T0 and T1 of the sample S10T increase to 298.7 °C and 345.5 °C at a level of 2.5 min^−1^, respectively, when compared to that of sample S10. During the process of surface treatment, the hemicelluloses were removed from the MS by using alkali; and a chemical reaction and physical tangle occurred between the MS and the silane, resulting in an improvement of the thermal stability. A similar effect of alkali/silane treatment on the thermal stability of biomass has been reported in other studies [35,36].

Although the thermal stability of the composites was enhanced by the alkali/silane treatment, it is still lower than that of PLA. After the treatment with alkali, the main content of MS is cellulose and lignin. The degradation temperature of lignin is in the range of 160–900 °C. Therefore, the thermal stability of the lignin containing MS/PLA composites will be lower than that of the pure PLA.

### 3.5. The Melt Flow Index of the Pure PLA and Its Composites

**Figure 6:**
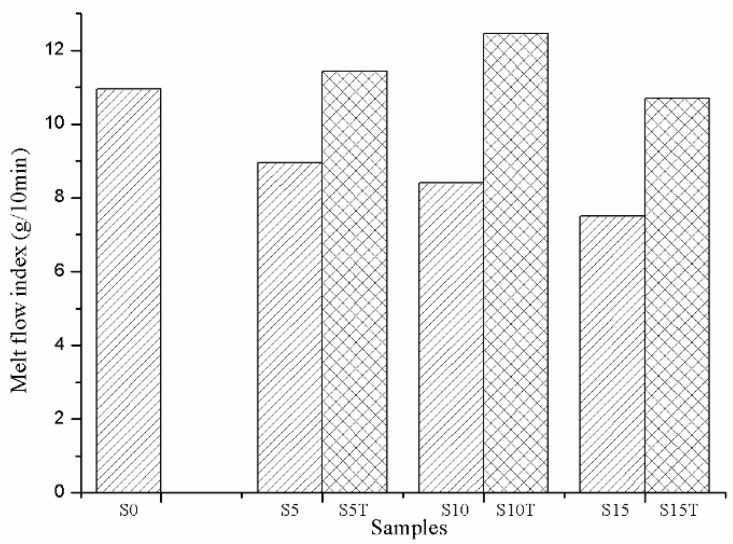
The Melt flow index of PLA and its composites.

Figure 6 shows the melt flow index (MFI) of the PLA/MS composites. When compared to that of the PLA (10.95 g/10 min), the MFI of the PLA/MS was reduced. The peclin and impurities contained in the MS led to the poor interface compatibility between the PLA matrix and the nutshell particles. Therefore, the MS particles interfered in the continuity of the matrix and hampered the heat transfer. With the increase of the content of the MS, the MFI of the PLA/MS decreases from 8.96 g/10 min (S5) to 7.51 g/10 min (S15). More MS meant more impurities containing, which would aggravate the interface incompatibility. Even more, a mass of MS particles had a strong tendency to aggregate, which hampered the heat transfer and lowered MFI.

The MFI of the PLA/MS composites were enhanced dramatically after the MS was treated by alkali/silane. The MFI of S10T leaps to the highest point with a value of 12.45 g/10 min, which together with the MFI of S5T (11.42 g/10 min) are even higher than that of the PLA. The hemicelluloses and impurities had been removed from MS through alkali treatment, resulting in a better interface compatibility. The siloxane acted as a bridge between the PLA and MS, leading to the rearrangement and repacking of the PLA molecules. Those all resulted in the improvement of the MFI of the composites.

### 3.6. The Morphological Structures

**Figure 7:**
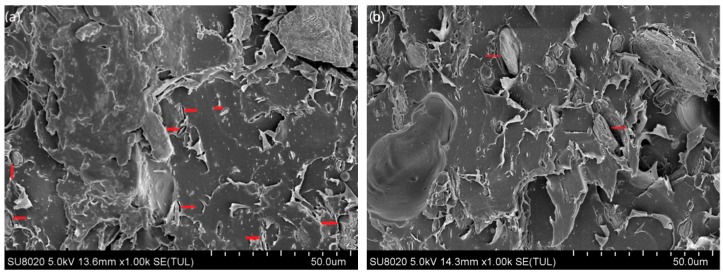

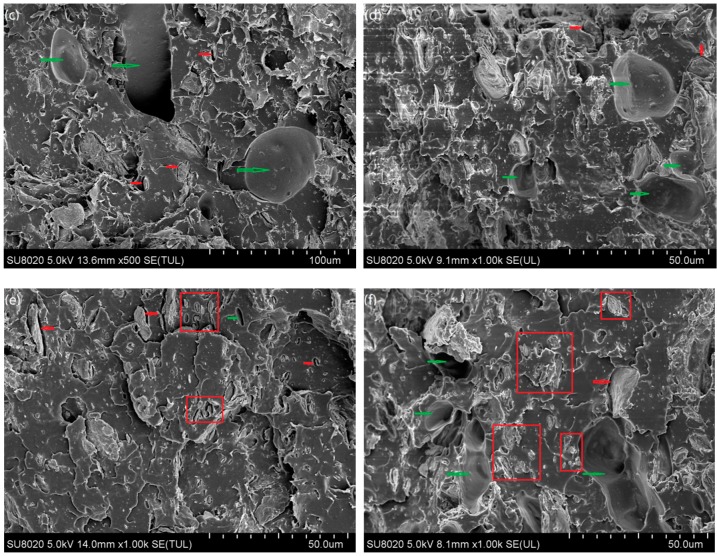
SEM micrographs of sample (**a**) S5, (**b**) S5T, (**c**) S10, (**d**) S10T, (**e**) S15 and (**f**) S15T.

**Figure 8:**
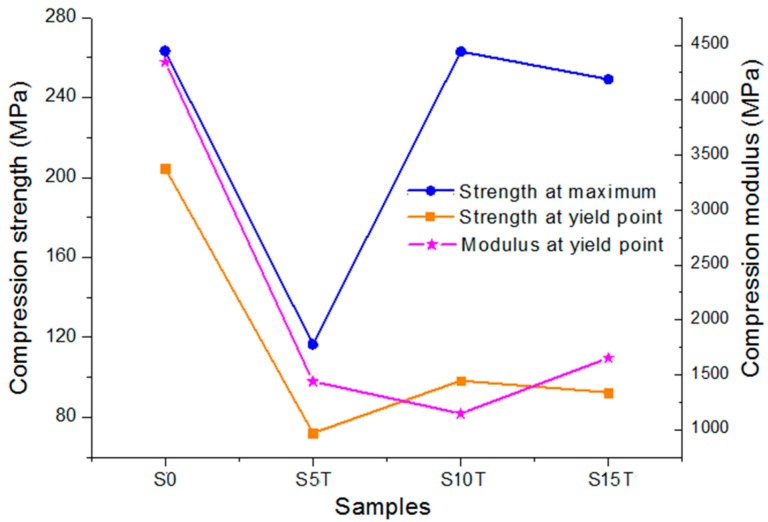
The relationship between the samples and the compressive properties of the PLA/treated MS composites.

**Figure 9:**
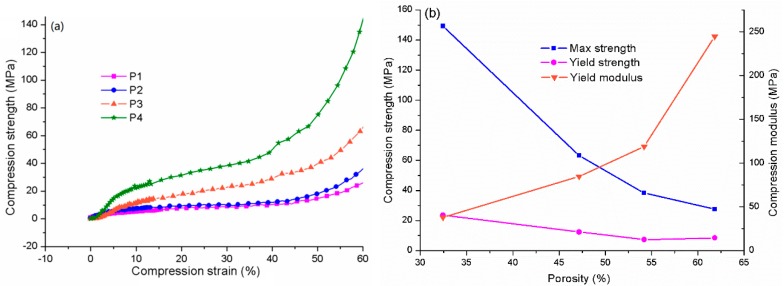
The compression relationship of PLA/MS composites between. (**a**) strength and strain and (**b**) the porosity and properties.

**Figure 10:**
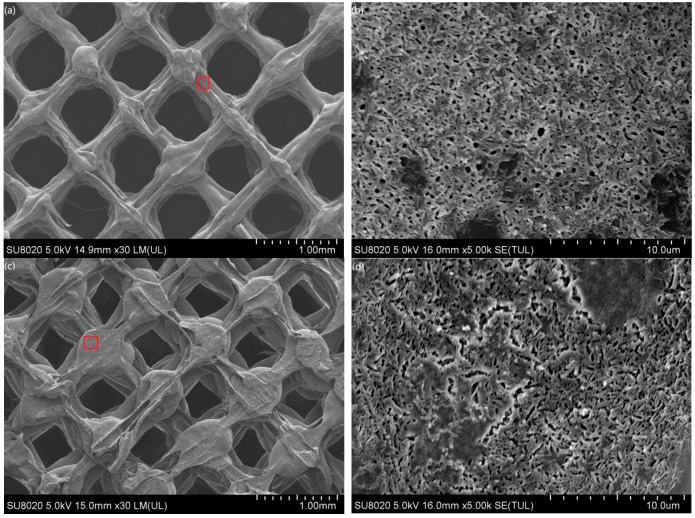

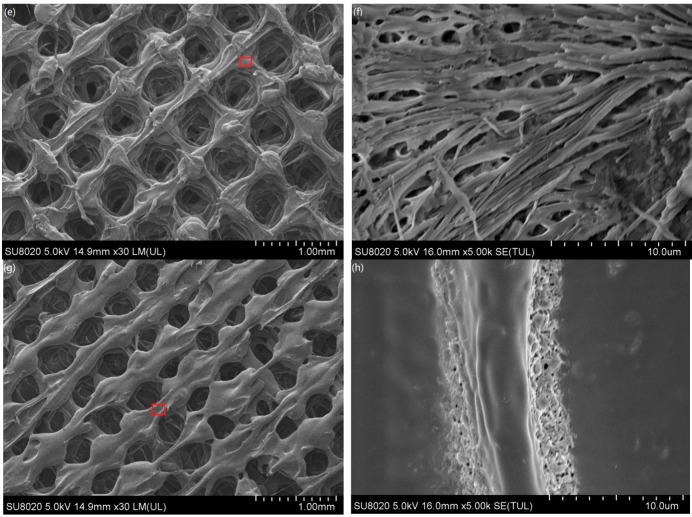
The morphologies of the PLA/MS composites scaffolds: (**a**) P1, (**b**) enlarger of red rectangle in P1, (**c**) P2, (**d**) enlarger of red rectangle in P2, (**e**) P3, (**f**) enlarger of red rectangle in P3, (**g**) P4 and (**h**) enlarger of red rectangle in P4.

Figure 7 illustrates the morphological structure of the PLA/MS composites. Compared to that of the untreated composites, the interfacial compatibility between the MS and the PLA matrix in treated composites was improved dramatically. There is an obvious clearance (red arrow) between the untreated MS and the PLA matrix, showing a poor interfacial compatibility. The number of this clearance is more aggravated with the increase of untreated MS content. The clearance would hamper the stress transfer during the mechanical tests. Some voids (green arrow), in size of 25–45 μm, due to the fiber pull-out, are also found on the S10, S10T and S15T samples regardless of the chemical treatment. These voids would become weak zones and weaken the load capacity of composites, and then further lower the strength level [36]. The size of voids in Figure 7d comparatively decreased when compared to that in Figure 7c to 10–28 μm for treated 10 wt% MS composite. When the ratio of the untreated MS in composites reaches to 15 wt%, some aggregations happened. From the red rectangle in Figure 7e, it can be seen that several MS particles are gathering in a small area. This strongly affected the adhesion between the MS and the PLA matrix. Although the aggregations still existed in ST15, its aggregation degree had reduced dramatically, and the treated particles’ adhesion in matrix was much stronger than the untreated ones.

### 3.7. The Compressive Properties

According to the previous analysis, the treated MS composites had a better thermal stability and fractural morphology than the untreated ones, so that the former was chosen for carrying out compressive tests. Figure 8 and Table 2 show the compression testing results. At the yield point, PLA possessed the highest strength and modulus of 204.7 MPa and 4346.1 MPa, respectively. However, the incorporation of the MS dramatically decreased both the yield strength and the modulus of the composites by more than 50%. The result showed that the stiffness of the composites declined. All the mechanical values of composites plunged to the bottom at the 5 wt% MS (S5T), indicating that the interconnection between the MS molecular and the PLA matrix was considerably loose. It can be confirmed by Figure 7b where there was a clearance between the PLA matrix and the MS particle. Similar results happened at the injection molded PLA/MS composites, where the tensile was less than half of that of the pure PLA [16]. Another 3D printed ABS/MS composite had about one third of strength of that of the ABS [21]. The strength at the maximum of the composites then climbed significantly again from the bottom (116.5 MPa at the S5T) to the climax (263.1 MPa at the S10T), and then decreased slightly to 249.2 MPa (at the S15T). The yield strength increased moderately from 72.1 MPa (S5T) to 98.3 MPa (S10T), following with a slightly drop to 92.4 MPa (S15T). The yield modulus of composites performed contrary to the strength, indicating that the stiffness of the composites was enhanced at a higher amount of MS. This result showed that a suitable content of the MS was able to fill tightly the gap between the PLA molecules (Figure 7d), and the redundant fillers would weaken the composites because of the aggregation (Figure 7e).

It is obvious that the dropping degree of the strength from S10T to S15T is only about one tenth of the strength from S0 to S5T, indicating that there is a possibility to manufacture additively the high content MS composites with an acceptable loading capacity. Meanwhile, the yield modulus of S15T is higher than that of S10T, showing that the higher content of MS is beneficial for improving the stiffness of the composites.

### 3.8. The Fabrication of Porous Scaffolds with S10T

#### 3.8.1. The Compression Properties of Scaffolds

It was proved previously that S10T has optimum thermal and compression properties, so this was chosen to fabricate the porous scaffolds. Table 3 and Figure 9 show the compression properties of the scaffolds with various pores and porosities. It can be seen from Figure 9a, all the curves experience three stages, including elastic deformation, yield and densification. Each scaffold possesses a yield plateau, indicating the scaffold acted as a buffer and absorbed the compressive energy when under pressure. Scaffolds with a larger porosity have a longer yield plateau, showing a stronger anti-deformation capability.

Figure 9b gives the relationship between the porosity and the compression properties. The max strength decreases rapidly with the increase of the porosity. The yield strength goes downwards steadily with a little rising in the end. These results illustrated that the load capability of the scaffolds was negatively affected by the porosity. However, it is noticed that the reduction of the max strength from P3 to P1 is more than one half of that from P4 to P3, showing a well stiffness at larger porosity. In contrast to the strength, the modulus climbs with the rising of the porosity, indicating that the stiffness was enhanced. The stiffness shows that the scaffolds have potential to be used as porous structural components.

#### 3.8.2. The Macro and Microstructure of Scaffolds

The macro and microstructures of scaffolds manufactured with PLA/MS are shown in Figure 10. The scaffold consists of beams and holes. The holes are in the shape of an irregular rectangle and are interconnected with each other. The length of those holes is in the range of 0.3–0.5 mm and is increasing with the rise of their porosity. The beam has a width of 0.2–0.5 mm. The size of the holes in the scaffold can be adjusted through adjusting the fill ratio of the FDM setup. The size of the micro pores can be controlled by changing the extrusion ratio of the FDM machine. In this way, a scaffold with various morphologies was obtained. The surfaces of P1 (Figure 10b) and P2 (Figure 10d) are porous and have pores of 0.1–1 μm in diameter. Figure 10f shows an obvious orientation with some pores between the printing lines. The pores almost disappear from the surface of P4 and distribute only along the sides of beam. The scaffolds in this work have porosity in a range of 30–62%, interconnected holes in size of 300–500 μm and micro pores in size of 0.1–1 μm. They can be used as porous structural components.

## 4. Conclusions

In this paper, the Macadamia nutshell (MS) filled PLA composites with or without NaOH and silane were fused deposition modeled (FDM) and characterized. The crystallinity, thermal stability, morphology and compression properties were compared and analyzed. 

The evaluation of the crystalline shows that the XRD intensity of NaOH treated MS was enhanced but was reduced after further treatment with silane. MS treated with 6 wt% silane had the highest crystalline among those treated MS. The thermal results showed that the PLA/treated MS composites were more stable than those untreated. S10T possessed the highest onset temperature of degradation (T_0_) and temperature at maximum degradation rate (T_1_) of 298.7 °C and 345.5 °C, respectively. The testing of the tensile properties showed that the yield strength and max strength of S10T reached a peak with values of 98.25 MPa and 263.08 MPa, respectively.

Finally, with S10T, the scaffolds with various porosities in a range of 30–65%, interconnected holes in size of 0.3–0.5 mm, micro pores with dimension of 0.1–1 μm and elastic modulus of 37.92–244.46 MPa were fabricated. The results indicated that the FDM of PLA/MS composites have the potential to be used as weight-lighter and structural components.

## Figures and Tables

**Table 1:** The formulations of the PLA/MS composites.

Samples Identification	PLA (wt%)	Untreated MS (wt%)	Treated MS (wt%)
S0	100	0	–
S5	95	5	–
S10	90	10	–
S15	85	15	–
S5T	95	–	5
S10T	90	–	10
S15T	85	–	15

**Table 2:** The compressive values of the PLA/treated MS composites.

Samples	Strength at the Maximum	Strength at Yield Point	Modulus at Yield Point
S0	263.4 ± 8.5	204.7 ± 3.6	4346.1 ± 68.6
S5T	116.5 ± 2.6	72.1 ± 2.4	1438.52 ± 31.5
S10T	263.1 ± 6.4	98.3 ± 3.2	1146.59 ± 53.2
S15T	249.2 ± 5.3	92.4 ± 2.1	1653.59 ± 35.6

**Table 3:** The porosity and compression properties of PLA/MS scaffolds.

Sample	Porosity (%)	Max Strength (MPa)	Yield Strength (MPa)	Yield Modulus (MPa)
P1	61.82	27.59	8.44	244.46
P2	54.19	38.28	7.33	118.88
P3	47.15	63.19	12.34	84.64
P4	32.44	149.31	23.65	37.92
